# β-Amyloid impairs Proteasome structure and function. Proteasome activation mitigates amyloid induced toxicity and cognitive deficits

**DOI:** 10.1101/2024.10.23.619877

**Published:** 2024-10-25

**Authors:** Kanisa Davidson, Mehar Bano, Danitra Parker, Pawel Osmulski, Maria Gaczynska, Andrew M. Pickering

## Abstract

**Background:**

Alzheimer’s Disease (AD) is the leading cause of dementia globally, affecting around 50 million people and marked by cognitive decline and the accumulation of β-amyloid plaques and hyperphosphorylated tau. The limited treatment options and numerous failed clinical trials targeting β-amyloid (Aβ) highlight the need for novel approaches. Lowered proteasome activity is a consistent feature in AD, particularly in the hippocampus. Impaired proteasome function in AD is hypothesized to stem from direct inhibition by β-amyloid or hyperphosphorylated tau, disrupting critical neuronal processes such as memory formation and synaptic plasticity.

**Objectives:**

This study tests the hypothesis that AD related deficits are driven in part by impaired proteasome function as a consequence of inhibition by Aβ. We evaluated how proteasome function is modulated by Aβ and the capacity of two proteasome-activating compounds, TAT1-8,9-TOD and TAT1-DEN to rescue Aβ-induced impairment in vitro, as well as survival deficits in cell culture and Aβ-induced cognitive deficits in Drosophila and mouse models.

**Results:**

Our study demonstrates that oligomeric β-amyloid binds to the 20S proteasome and impairs its activity and conformational stability. The oligomers also destabilize the 26S proteasome to release the free 20S proteasome. Treatment with proteasome activators TAT1-8,9TOD and TAT1-DEN rescue the 20S proteasome function and reduces cell death caused by Aβ42 toxicity in SK-N-SH cells. In Drosophila models overexpressing Aβ42, oral administration of proteasome agonists delayed mortality and restored cognitive function. Chronic treatment with TAT1-DEN protected against deficits in working memory caused by Aβ42 in mice and in hAPP(J20) mice with established deficits, acute TAT1-DEN treatment significantly improved spatial learning, with treated mice performing comparably to controls.

**Conclusions:**

Aβ has dual impacts on 20S and 26S proteasome function and stability. Proteasome activation using TAT1-8,9TOD and TAT1-DEN shows promise in mitigating AD-like deficits by protecting against amyloid toxicity and enhancing proteasome function. These findings suggest that targeting proteasome activity could be a viable therapeutic approach for AD, warranting further investigation into the broader impacts of proteasome modulation on AD pathology.

## Full Text Availability

The license terms selected by the author(s) for this preprint version do not permit archiving in PMC. The full text is available from the preprint server.

